# Mesenchymal Stromal Cells: Inhibiting PDGF Receptors or Depleting Fibronectin Induces Mesodermal Progenitors with Endothelial Potential

**DOI:** 10.1002/stem.1538

**Published:** 2014-02-19

**Authors:** S G Ball, J J Worthington, A E Canfield, C L R Merry, C M Kielty

**Affiliations:** a Wellcome Trust Centre for Cell-Matrix Research, School of Materials, Faculty of Engineering and Physical Sciences, University of ManchesterManchester, Lancashire, United Kingdom; b Faculty of Life Sciences, School of Materials, Faculty of Engineering and Physical Sciences, University of ManchesterManchester, Lancashire, United Kingdom; c Faculty of Medical and Human Sciences, and, School of Materials, Faculty of Engineering and Physical Sciences, University of ManchesterManchester, Lancashire, United Kingdom; d Stem Cell Glycobiology Group, School of Materials, Faculty of Engineering and Physical Sciences, University of ManchesterManchester, Lancashire, United Kingdom

**Keywords:** Mesenchymal stromal cells, Spheroids, Platelet-derived growth factor receptor, Fibronectin, Endothelial, Neovascularization

## Abstract

Realizing the full therapeutic potential of mesenchymal stromal/stem cells (MSCs) awaits improved understanding of mechanisms controlling their fate. Using MSCs cultured as spheroids to recapitulate a three-dimensional cellular environment, we show that perturbing the mesenchymal regulators, platelet-derived growth factor (PDGF) receptors or fibronectin, reverts MSCs toward mesodermal progenitors with endothelial potential that can potently induce neovascularization *in vivo*. MSCs within untreated spheroids retain their mesenchymal spindle shape with abundant smooth muscle α-actin filaments and fibronectin-rich matrix. Inhibiting PDGF receptors or depleting fibronectin induces rounding and depletes smooth muscle α-actin expression; these cells have characteristics of mesenchymoangioblasts, with enhanced expression of mesendoderm and endoderm transcription factors, prominent upregulation of E-cadherin, and Janus kinase signaling-dependent expression of Oct4A and Nanog. PDGF receptor-inhibited spheroids also upregulate endothelial markers platelet endothelial cell adhesion molecule 1 and vascular endothelial-cadherin and secrete many angiogenic factors, and *in vivo* they potently stimulate neovascularization, and their MSCs integrate within functional blood vessels that are perfused by the circulation. Thus, MSC potency and vascular induction are regulated by perturbing mesenchymal fate.

## Introduction

Fulfilling the potential of mesenchymal stromal (stem) cells (MSCs) in regenerative medicine requires an improved understanding of the mechanisms that control their fate. It is well known that, in adherent cultures, MSCs adopt a myofibroblast-like contractile phenotype and can differentiate along mesenchymal lineages in response to defined supplements 1,2. Less is known about MSCs *in vivo* and even the lineage specification of their embryonic precursors is ill-defined 3,4. MSCs occupy perivascular niches throughout the body 5 and in the bone marrow can undergo osteogenic differentiation and support hematopoiesis 6,7. Although MSCs can express endothelial markers *in vitro* in response to growth factors 8, or to cell density-dependent Notch signals 9, their ability to form functional vascular endothelium and contribute to new blood vessel formation *in vivo* remains uncertain. We report that MSC fate is changed by perturbing mesenchymal regulators, which in turn stimulates neovascularization and their integration into functional blood vessels.

MSCs are derived predominantly from the mesodermal lineage, but also from endoderm by epithelial-mesenchymal transition and from ectodermal neural crest 10–12. During development, the mesoderm forms distinct mesenchymal and hemato-endothelial lineages. Using embryonic stem cells directed toward mesendoderm, one group identified a common mesoderm-derived precursor for MSCs and endothelial cells, which they termed a mesenchymoangioblast 3,4. Others described a bone marrow mesodermal progenitor cell population with dual mesenchymal and endothelial differentiation potential 13. These data point to a mesodermal cell stage with potential to form mesenchyme or endothelium.

Platelet-derived growth factor (PDGF) receptors (PDGFR) are markers and critical regulators of mesenchyme 14–16. Knockout mice showed that loss of PDGFRα or PDGF-A disrupts mesenchymal tissue formation, whereas loss of PDGFRβ disrupts pericytes and smooth muscle 17,18. Knockout of PDGFRα caused death of 50% of embryos before E10 and the rest shortly after birth 19, while in chick, signaling through PDGFRα was required for mesodermal cell migration 20. We have shown that PDGFR signaling in MSCs regulates migration, proliferation, and cytoskeletal organization, through RhoA/Rho kinase (ROK) signaling 21 and by crosstalk with fibronectin (FN)-activated integrin α5β1 22 and neuropilin-1 23. We showed that FN/α5β1 activates PDGFRβ in the absence of PDGF growth factors, and is also required to potentiate PDGF-BB-mediated PDGFRβ activation 22. FN, a chordate innovation, is an extracellular adhesive glycoprotein 24, which controls the deposition of fibrillar matrices by mesenchymal cells 25, and thus tissue formation. FN-null mice are early embryonic lethal due to multiple cardiovascular defects 26. PDGFRβ signaling enhances FN expression 27, and together they are potent drivers of mesenchyme.

We have tested the hypothesis that disrupting mesenchymal regulators can alter the fate of human bone marrow-derived MSCs. Cell cytoskeleton was modified by inhibiting PDGFRs or by depleting FN, within three-dimensional (3D) spheroids. Resulting MSCs were rounded rather than spindle-shaped, with depleted smooth muscle α-actin (SMA) filaments and greatly reduced migratory capacity. They were mesenchymoangioblast-like with enhanced transcription factors such as EOMES, Foxh1, and Mixl1. These cells also exhibited marked upregulation of E-cadherin, Oct4A, and Nanog as well as endothelial markers platelet endothelial cell adhesion molecule 1 (PECAM-1) and vascular endothelial (VE)-cadherin and angiogenic growth factors. They had endothelial-like organization, and markedly enhanced neovascularization and integration into new functional blood vessels that were perfused by the circulation *in vivo*. Thus, perturbation of mesenchymal regulators modulates MSC fate and angiogenic potential *in vivo*. This discovery offers opportunities for therapeutic revascularization.

## Materials and Methods

### Cell Culture and Spheroid Formation

Human bone marrow-derived MSCs from a 21-year-old female, and 21- and 33-year-old males (Lonza, Allendale, NJ, http://www.lonza.com), were subcultured on 0.1% gelatin, maintained in MesenPRO RS growth medium (Life Technologies, Grand Island, NY, http://www.lifetechnologies.com), and used at passage 5. Spheroids were formed by seeding 60,000 MSCs in growth medium ± 0.1 µM PDGFR inhibitor-IV (EMD Millipore, Billerica, MA, http://www.emdmillipore.com) 28, into individual wells of a low cell binding 96-well plate (Nunc 145399; Thermo Scientific, Waltham, MA, http://www.thermofisher.com) and cultured at 37°C for 5 days. Other small molecular inhibitors; EGFR (PD168393), FGFR (341608), VEGFR (ZM323881), Rho-kinase (H-1152), Rac1 (553508), MEK (PD98059), PI3K (LY294002), and JAK (Inhibitor I), were all obtained from EMD Millipore, Billerica, MA, http://www.emdmillipore.com and previously described 28.

### Quantitative RT-PCR Analysis

For RNA isolation; ≥12 identically cultured spheroids were pooled together for analysis. RNA was isolated using Trizol reagent (Life Technologies, Grand Island, NY, http://www.lifetechnologies.com) followed by digestion with RNase-free DNase (Promega, Madison, WI, http://www.promega.com). First strand cDNA synthesis was performed using AMV reverse transcriptase (Roche, Basel, Switzerland, http://www.roche.com), and real-time quantitative PCR using GoTaq qPCR kit (Qiagen, Valencia, CA, http://www.qiagen.com). Gene expression was determined relative to GAPDH using the Δ*C*_t_ method. All primer sequences are provided in Supporting Information Table S1.

### siRNA Knockdown

MSCs were transfected with 10 nM small interfering RNAs (siRNAs) using Lipofectamine RNAi MAX reagent (Life Technologies, Grand Island, NY, http://www.lifetechnologies.com), then cultured for 24 hours in the presence of the reagent and siRNA. Transfected MSCs were then trypsinized and used to form spheroids in the presence of fresh Lipofectamine reagent and siRNA which remained in the culture at 37°C for 5 days. Validated siRNAs were used to knockdown FN (1027417) (Qiagen, Valencia, CA, http://www.qiagen.com); scrambled siRNA (Qiagen, Valencia, CA, http://www.qiagen.com) was used as a control.

### Immunoblotting

For protein isolation; ≥12 identically cultured spheroids were pooled together for analysis. Protein lysate isolation, immunoblotting, and quantification were performed as previously described 21. Details of the antibodies used are given in Supporting Information Table S2.

### Matrigel In Vitro Network and Implant Analysis

For network formation, spheroids were dissociated into single cells by incubation with 0.001% (wt/vol) collagenase type IV (Sigma Aldrich, St. Louis, MO, http://www.sigmaaldrich.com) in phosphate buffered saline for 1 hour at 37°C, followed by TrypLE Select (Life Technologies, Grand Island, NY, http://www.lifetechnologies.com) digestion for 10 minutes. MSCs were seeded onto growth factor reduced Matrigel (BD Biosciences, San Jose, CA, http://www.bdbiosciences.com) and incubated at 37°C. For implants, spheroids were covered with Matrigel and incubated at 37°C.

### Matrigel Plug In Vivo Angiogenesis Assay

Spheroids suspended in 200 µl growth medium and 500 µl of growth factor reduced Matrigel were injected subcutaneously into the flanks of 6–8-week-old C57BL/6 mice. Five mice were each injected with 10 control spheroids and five with 10 PDGFR-IV spheroids. Two weeks after injection, mice were sacrificed, plugs removed, and immediately fixed in 4% (wt/vol) paraformaldehyde for 24 hours at 4°C, then incubated in 30% (wt/vol) sucrose solution for 24 hours at 4°C. Plugs were then embedded in OCT and 10 µm sections obtained for immunofluorescence analysis which was performed as previously described 28.

To study whether new blood vessels were functional and integrated with the circulation, 200 µl of 50 mg/ml FITC-dextran 2,000 kDa (Sigma Aldrich, St. Louis, MO, http://www.sigmaaldrich.com) was injected into the tail vein of mice, and allowed to perfuse for 10 minutes before the mice were sacrificed, and the plugs excised and processed, as above.

### Whole Mount Immunofluorescence Analysis and Microscopy

Spheroids were prepared for confocal microscopy as described 29. Details of the antibodies used are given in Supporting Information Table S2. Images were collected on a Nikon C1 confocal using a TE2000 PSF inverted microscope, using ×60 /NA 1.40 Plan Apo or ×20/NA 0.50 Plan Fluor objectives and ×3 confocal zoom. Different sample images detecting the same antibodies were acquired under constant acquisition settings. Images were processed using Nikon EZ-C1 FreeViewer v3.3 software. Bright-field images were collected on an Olympus BX51 widefield microscope, using a ×10/NA 0.3 UPlan F1 objective. Images were captured with a CoolSNAP camera system and processed using MetaMorph imaging v5.0 software.

### Statistical Analysis

Results are expressed as the mean ± SD. Statistical differences between sets of data were determined using a paired Student's *t* test, with *p* < .05 considered significant.

## Results

PDGFRs regulate the formation of mesenchymal cells from mesoderm 14,16. These cells in turn make tissues by depositing and embedding themselves within a FN-rich extracellular matrix 24,25. FN/α5β1 is functionally linked with PDGFRβ, both by activating PDGFRβ in the absence of PDGF growth factors and by potentiating PDGF-BB-induced PDGFRβ signaling 22. Here, we used 3D spheroid cultures to test the hypothesis that cytoskeletal changes induced by inhibiting these functionally integrated mesenchymal regulators, PDGFR signaling and FN, revert MSCs toward a mesodermal progenitor with endothelial potential.

### PDGFR Signaling Modulates MSC Spheroid Assembly and Organization

**Figure 1:**
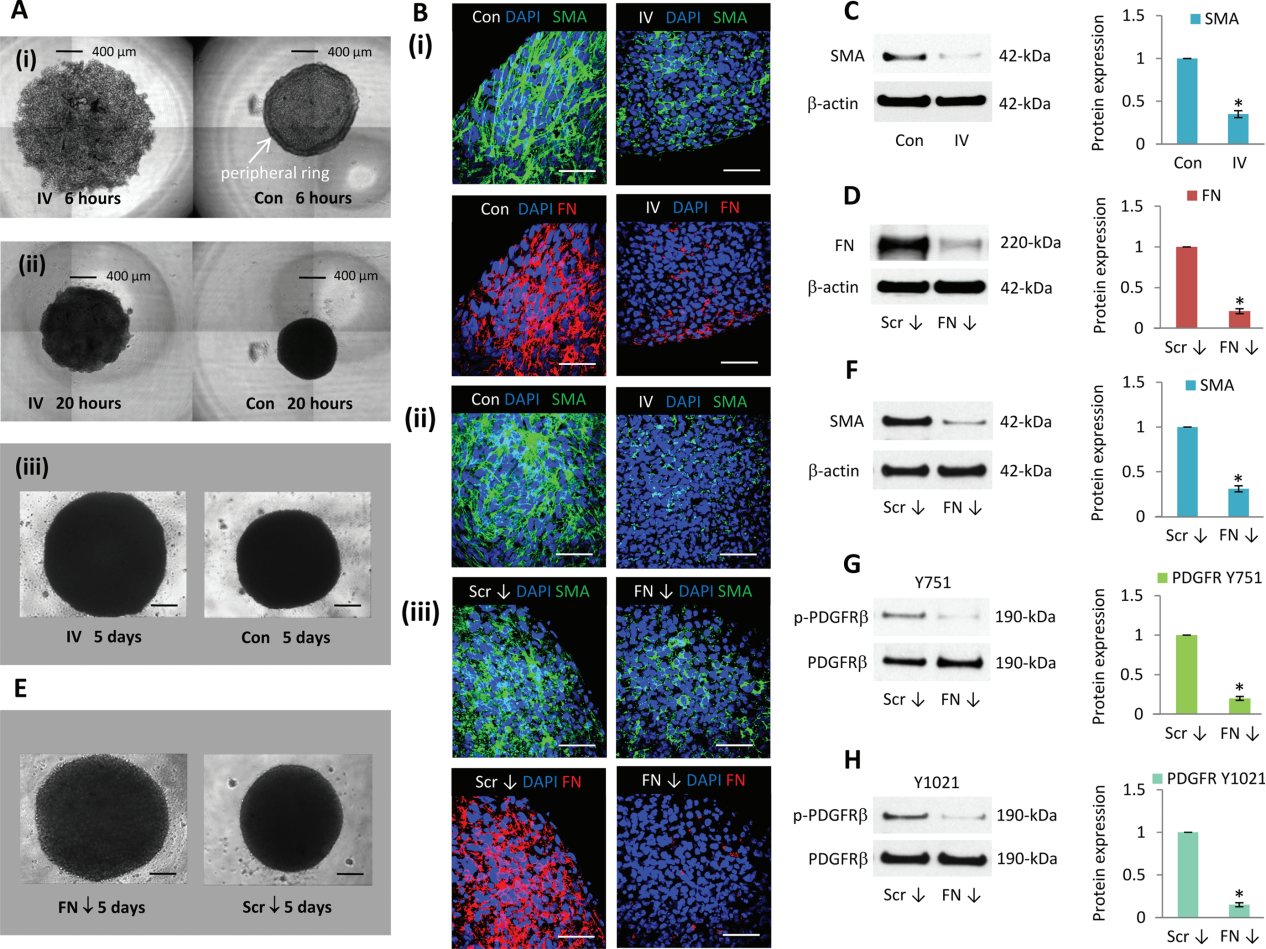
PDGFR and FN inhibition changed mesenchymal stromal/stem cell (MSC) spheroid shape and SMA expression. (A): Bright-field images of control and PDGFR inhibitor-IV treated MSCs during spheroid assembly, showing cells (i) 6 hours, (ii) 20 hours, and (iii) 5 days following seeding. Scale bars = 400 µm (i, ii) and 200 µm (iii). (B): Whole mount immunofluorescence analysis of (i, ii) control spheroids (con) and PDGFR inhibitor-IV spheroids (IV), (iii) scrambled control siRNA spheroids (Scr ↓) and FN knockdown spheroids (FN ↓) cultured for 5 days, showing FN (red) and SMA (green) expression, with DAPI-stained nuclei (blue). Scale bars = 50 µm. (C): Immunoblot analysis of SMA expression within control (Con) and PDGFR inhibitor-IV (IV) spheroids after 5 days culture, with β-actin as a loading control. Histogram shows SMA expression relative to β-actin and normalized to control spheroid level. *, *p* < .001 compared with day 5 control spheroids, using paired *t* test *n* = 3 separate experiments, error bars represent SD. (D): Immunoblot analysis of FN expression within control scrambled siRNA spheroids (Scr ↓) and FN knockdown spheroids (FN ↓) cultured for 5 days, with β-actin as a loading control. RNA expression is relative to GAPDH and normalized to the level of scrambled control spheroids at day 5. Histogram shows protein expression relative to β-actin and normalized to control siRNA spheroid level. *, *p* < .001 compared with control siRNA spheroids, using paired *t* test *n* = 3 separate experiments, error bars represent SD. (E): Bright-field images of spheroids assembled using MSCs treated with scrambled (Scr) or FN small interfering RNAs (siRNA) and cultured for 5 days. Scale bars = 200 µm. (F–H): Immunoblot analysis of SMA, PDGFRβ Y751, and Y1021 phosphorylation levels, within scrambled control siRNA spheroids (Scr ↓) and FN knockdown spheroids (FN ↓) after 5 days culture, with β-actin or PDGFRβ as a loading control. Histograms show SMA expression relative to β-actin and PDGFRβ Y751 and Y1021 relative to PDGFRβ, normalized to scrambled control siRNA spheroid level. *, *p* < .001 compared with control spheroids, using paired *t* test *n* = 3 separate experiments, error bars represent SD. Abbreviations: FN, fibronectin; PDGFR, platelet-derived growth factor receptor.

PDGFR signaling regulates the expression of contractile SMA filaments 14–16, which are not only a characteristic functional feature of vascular smooth muscle cells 30 but also abundantly expressed by MSCs 21, and are widely used as a distinctive mesenchyme marker. Using live cell imaging, we monitored the assembly of MSCs into control and PDGFR inhibitor-IV spheroids during the first 20 hours of formation (Supporting Information Video S1). While control MSC suspensions rapidly aggregated to form a spheroid, their assembly in the presence of PDGFR inhibitor-IV was markedly slower. After 6 hours of seeding, control MSCs formed a spheroid-like structure which contains a distinct outer ring of cells (Fig. 1A(i)); this feature was not detected during the assembly of PDGFR inhibitor-IV spheroids. After 20 hours of seeding, control MSCs formed a spherical structure (Fig. 1A(ii)), whereas PDGFR inhibitor-IV spheroids had a larger diameter but more flattened structure. These distinctive organizational features were maintained throughout a 5-day culture period (Fig. 1A(iii)); MSCs within control 3D spheroids retained their characteristic spindle-shaped morphology and expressed widespread abundant extended SMA filaments and FN (Fig. 1B(i)). However, MSCs within PDGFR inhibitor-IV spheroids were more rounded, and SMA and FN abundance were markedly reduced (Fig. 1B(i)). Within the core of the spheroids, MSCs appeared viable and expressed abundant SMA filaments (Fig. 1B(ii)), which were diminished by PDGFR inhibitor-IV. Compared to control spheroids at day 5, PDGFR inhibitor-IV spheroids expressed 2.9 ± 0.3-fold lower SMA protein (Fig. 1C).

PDGFRβ is known to regulate FN expression 27. Having shown that PDGFR inhibitor-IV spheroids contained markedly reduced SMA and FN, we used siRNA knockdown to investigate how FN influences the fate of MSCs within 3D spheroids. Compared to spheroids formed from scrambled siRNA knockdown MSCs, spheroids assembled by FN knockdown MSCs displayed a 79% ± 9% reduction in FN protein (Fig. 1D) expression, respectively, by day 5 of culture. Like the PDGFR inhibitor-IV treated spheroids (Fig. 1A), these FN knockdown spheroids had larger diameters than control spheroids (Fig. 1E). The FN knockdown MSCs also appeared more rounded and in closer contact than control cells and, compared to scrambled siRNA knockdown spheroids, they expressed markedly reduced SMA filaments (Fig. 1B(iii)) and had a 70% ± 8% decrease in SMA protein expression (Fig. 1F). FN knockdown spheroids also displayed significantly diminished PDGFRβ phosphorylation (Y751 80% ± 7% and Y1021 85% ± 8% reduction, respectively) (Fig. 1G, 1H). These data show that PDGFRs, and FN which activates PDGFRs, both regulate SMA contractile filaments and coordinate MSC spheroid organization and contractility.

### PDGFR Inhibition and FN Depletion Increase Oct4A and Nanog Expression

**Figure 2:**
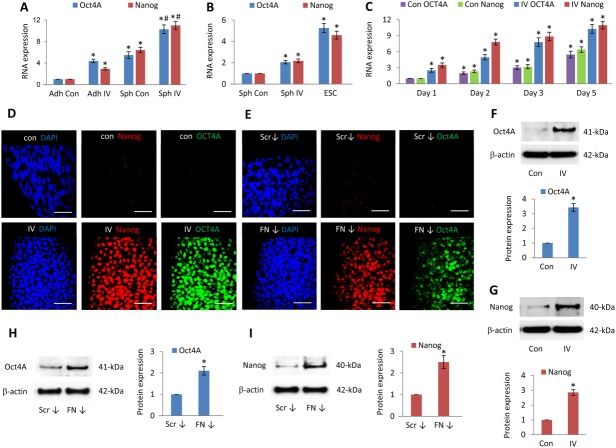
Platelet-derived growth factor receptor (PDGFR) and fibronectin (FN)-inhibited spheroids upregulate Oct4A and Nanog. (A): Quantitative RT-PCR analysis of Oct4A and Nanog expression within high-density adherent mesenchymal stromal/stem cells (MSCs) (Adh Con), high-density adherent MSCs exposed to PDGFR inhibitor-IV (Adh IV), control spheroids (Sph Con), and PDGFR inhibitor-IV spheroids (Sph IV), cultured for 5 days. Data are relative to GAPDH and normalized to adherent control levels. *, *p* < .001 compared with adherent controls, ^#^, *p* < .001 compared with control spheroids, using paired *t* test *n* = 3 separate experiments, error bars represent SD. (B): Quantitative RT-PCR analysis of Oct4A and Nanog expression within control spheroids (Sph Con) and PDGFR inhibitor-IV spheroids (Sph IV), cultured for 5 days, and human embryonic stem cell line Hues1. Data are relative to GAPDH and normalized to control spheroid levels. *, *p* < .001 compared with control spheroids, using paired *t* test *n* = 2 separate experiments, error bars represent SD. (C): Quantitative RT-PCR analysis of Oct4A and Nanog expression within control spheroids (Con) and PDGFR inhibitor-IV spheroids (IV), cultured for cultured for 1, 2, 3, and 5 days. Data are relative to GAPDH and normalized to the level of control spheroids at day 1. *, *p* < .001 compared with day 1 control spheroids, using paired *t* test *n* = 3 separate experiments, error bars represent SD. (D, E): Whole mount immunofluorescence analysis of (D) control spheroids (Con) and PDGFR inhibitor-IV spheroids (IV), (E) scrambled control siRNA spheroids (Scr ↓), and FN knockdown spheroids (FN ↓) cultured for 5 days, showing Nanog (red) and Oct4A (green) expression, with DAPI-stained nuclei (blue). Scale bars = 50 µm. (F–I): Immunoblot analysis of Oct4A and Nanog expression within (F, G) control (Con) and PDGFR inhibitor-IV (IV) spheroids and (H, I) scrambled control siRNA spheroids (Scr ↓) and FN knockdown spheroids (FN ↓) after 5 days culture, with β-actin as a loading control. Histograms show Oct4A and Nanog expression relative to β-actin and normalized to control spheroid or scrambled control siRNA control spheroid levels. *, *p* < .001 compared with control spheroids, using paired *t* test *n* = 3 separate experiments, error bars represent SD.

Having shown that PDGFR inhibition and FN knockdown altered MSC organization within 3D spheroids, we determined their effects on expression of the pluripotency markers Oct4A and Nanog. Compared to untreated adherent MSCs, control spheroids increased Oct4A (5.5 ± 0.6-fold) and Nanog (6.4 ± 0.5-fold) transcript levels, and PDGFR inhibitor-IV spheroids further increased Oct4A (10.4 ± 0.8-fold) and Nanog (11.0 ± 0.7-fold) (Fig. 2A). To compare these transcript levels with those expressed by pluripotent human embryonic stem cells (ESCs), we examined levels of Oct4A and Nanog expressed by a human ESC line Hues1 (Fig. 2B). Compared to control spheroids, PDGFR inhibitor-IV spheroids increased Oct4A (2.1 ± 0.2-fold) and Nanog (2.2 ± 0.2-fold), while ESCs demonstrated a greater level of Oct4A (5.3 ± 0.4-fold) and Nanog (4.6 ± 0.4-fold). Oct4A and Nanog transcript expression increased throughout the 5-day culture in both control and PDGFR inhibitor-IV-treated spheroids, with the PDGFR inhibitor-IV spheroids producing the greater increase (Fig. 2C).

Immunofluorescence analysis revealed no Oct4A and a low abundance of Nanog within day 5 control spheroids (Fig. 2D). In contrast, Nanog and Oct4A were initially detected at day 3 and day 1, respectively, in PDGFR inhibitor-IV spheroids (Supporting Information Fig. S1), and both factors increased in abundance up to day 5 (Fig. 2D). Both Nanog and Oct4A expression were dependent on Janus kinase (JAK) signaling (Supporting Information Fig. S2A, S2B), while nuclear STAT3 (Y705) and STAT1 (Y701) increased markedly within PDGFR inhibitor-IV spheroids (Supporting Information Fig. S2C). Compared to control spheroids at day 5, PDGFR inhibitor-IV spheroids expressed significantly higher levels of both Oct4A (3.3 ± 0.3-fold) (Fig. 2F) and Nanog (3.0 ± 0.2-fold) protein (Fig. 2G), which were JAK signaling-dependent (Supporting Information Fig. S2D).

Immunofluorescence analysis revealed that, while there was no detectable Oct4A, and low Nanog expression within day 5 scrambled knockdown spheroids, FN knockdown spheroids markedly increased Nanog and Oct4A expression (Fig. 2E). Immunoblot analysis also confirmed an increase in Oct4A (2.1 ± 0.2-fold) (Fig. 2H) and Nanog (2.5 ± 0.3-fold) (Fig. 2I), although these levels were lower than in the PDGFR inhibitor-IV spheroids.

Thus, in comparison to adherent MSC cultures, spindle-shaped MSCs within 3D control spheroids increased the expression of Oct4A and Nanog transcripts, although only low levels of protein were detected. However, inhibition of PDGFR signaling or FN knockdown within spheroids, which induced more rounded MSCs, prominently upregulated Oct4A and Nanog protein levels.

### MSC Spheroids Display a Mesendoderm Expression Profile

**Figure 3:**
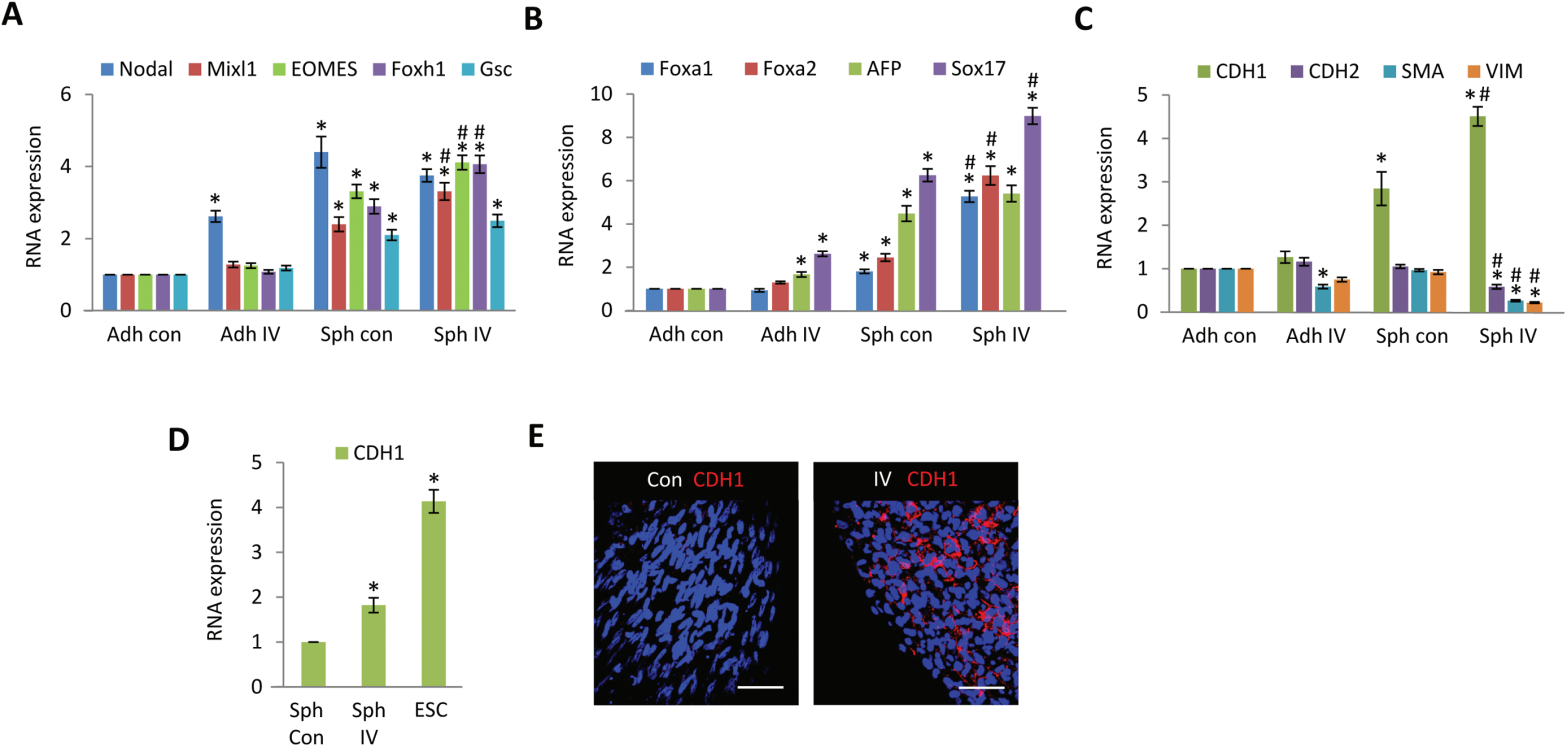
Platelet-derived growth factor receptor (PDGFR)-inhibited spheroids express E-cadherin. (A–C): Quantitative RT-PCR analysis of (A) Nodal, Mixl1, EOMES, Foxh1, and Goosecoid (Gsc) (B) Foxa1, Foxa2, AFP, and Sox17, (C) E-cadherin (CDH1), N-cadherin (CDH2), SMA, and vimentin (VIM), within high-density adherent mesenchymal stromal/stem cells (MSCs) (Adh con), high-density adherent MSCs exposed to PDGFR inhibitor-IV (Adh IV), control spheroids (Sph con), and PDGFR inhibitor-IV spheroids (Sph IV), cultured for 5 days. Data are relative to GAPDH and normalized to adherent control levels. *, *p* < .001 compared with adherent controls, ^#^, *p* < .005 compared with control spheroids, using paired *t* test *n* = 3 separate experiments, error bars represent SD. (D): Quantitative RT-PCR analysis of CDH1 within control spheroids (Sph Con) and PDGFR inhibitor-IV spheroids (Sph IV), cultured for 5 days, and human ESC line Hues1. Data are relative to GAPDH and normalized to control spheroid levels. *, *p* < .001 compared with spheroid controls, using paired *t* test *n* = 2 separate experiments, error bars represents SD. (E): Whole mount immunofluorescence analysis of control spheroids (con) and PDGFR inhibitor-IV spheroids (IV) cultured for 5 days, showing CDH1 (red) expression with DAPI-stained nuclei (blue). Scale bars = 50 µm. Abbreviations: AFP, α-fetoprotein; ESC, embryonic stem cell.

Having established that MSCs cultured as 3D spheroids markedly upregulate the expression of the pluripotency transcription factors Nanog and Oct4A, we investigated expression levels of mesendoderm and endoderm transcription factors expressed by spheroids at day 5 (Fig. 3). Bipotent mesendoderm cells are precursors for both endoderm and mesoderm during ESC differentiation 31. Mesendoderm has been characterized as goosecoid (Gsc)^+^ E-cadherin (CDH1)^+^ PDGFRα^+^ which can give rise to Gsc^+^ CDH1^+^ PDGFRα^−^ endoderm and Gsc^+^ CDH1^−^ PDGFRα^+^ mesoderm progenitors 31. Compared to untreated adherent MSCs, control spheroids expressed increased transcripts of mesendoderm transcription factors; Nodal, Mixl, EOMES, Foxh1, and Gsc (Fig. 3A), but PDGFR inhibitor-IV spheroids expressed higher levels of Mixl, EOMES, and Foxh1 (Fig. 3A). Control spheroids also expressed increased levels of the endoderm transcription factors; Foxa1, Foxa2, α-fetoprotein (AFP), and Sox17 (Fig. 3B), which were further increased in PDGFR inhibitor-IV spheroids (Fig. 3B).

We therefore examined the expression of the definitive endoderm marker CDH1 32, which is essential for maintaining ESC pluripotency 33 and is implicated in iPS cell generation 34. Compared to untreated adherent MSCs, control spheroids expressed increased CDH1 (2.8 ± 0.4-fold) transcript; however, levels of mesoderm markers CDH2, SMA, and vimentin remained similar (Fig. 3C). In contrast, PDGFR inhibitor-IV spheroids expressed significantly higher CDH1 (4.5 ± 0.2-fold), whereas levels of CDH2, SMA, and vimentin were markedly decreased (Fig. 3C). Compared to control spheroids, PDGFR inhibitor-IV spheroids showed an increase of 1.8 ± 0.1-fold in CDH1 transcript, while human ESCs demonstrated a higher level (4.1 ± 0.3-fold) (Fig. 3D). Immunofluorescence analysis revealed no detectable CDH1 protein within control spheroids (Fig. 3E), but CDH1 protein was readily detected and characteristically localized at cell-cell boundaries within PDGFR inhibitor-IV spheroids (Fig. 3E).

To investigate further the increased expression of pluripotency and endoderm proteins within PDGFR inhibitor-IV spheroids, a stem cell proteome array was used to detect relative expression levels simultaneously (Supporting Information Fig. S3A). Compared to control spheroids, PDGFR inhibitor-IV spheroids showed a distinct increase in the pluripotent markers; Oct4, Nanog, and Sox2, and endoderm markers; Sox17, AFP, Foxa2, and CDH1. These data verify that PDGFR inhibitor-IV spheroids express mesendoderm and endoderm markers including CDH1, indicating features of a mesendoderm progenitor.

Proteome array analysis demonstrated that, compared to scrambled siRNA knockdown spheroids, FN knockdown spheroids also upregulated Oct4, Nanog, and Sox2 as well as endoderm markers; Sox17, AFP, Foxa2, and CDH1 (Supporting Information Fig. S3B). Thus, FN knockdown also resulted in features of a mesendoderm progenitor, although the relative levels of these markers were lower than those induced by PDGFR inhibition (Supporting Information Fig. S3A). These data indicate that inhibition of PDGFR signaling, or FN depletion, within spheroids reverts MSCs toward a more multipotent state.

### MSC Spheroids Exhibit Endothelial Characteristics

Using mesoderm progenitor cells derived from ESCs, activation of PDGFRβ signaling can induce differentiation to vascular smooth muscle cells, which express abundant SMA, whereas stimulation with VEGF-A can induce differentiation toward endothelial cells 35. In addition, adherent MSCs cultured in two-dimensional (2D) at high density acquire a more rounded cobblestone-like morphology, and can undergo differentiation toward an endothelial lineage 9. We therefore explored the possibility that MSCs within PDGFR inhibitor-IV spheroids, which lack PDGFR signaling, have depleted SMA and are at high density, may exhibit an endothelial rather than mesenchymal disposition. Accordingly, the expression of two characteristic endothelial markers, PECAM-1 and VE-cadherin, was determined in PDGFR inhibitor-IV spheroids.

**Figure 4:**
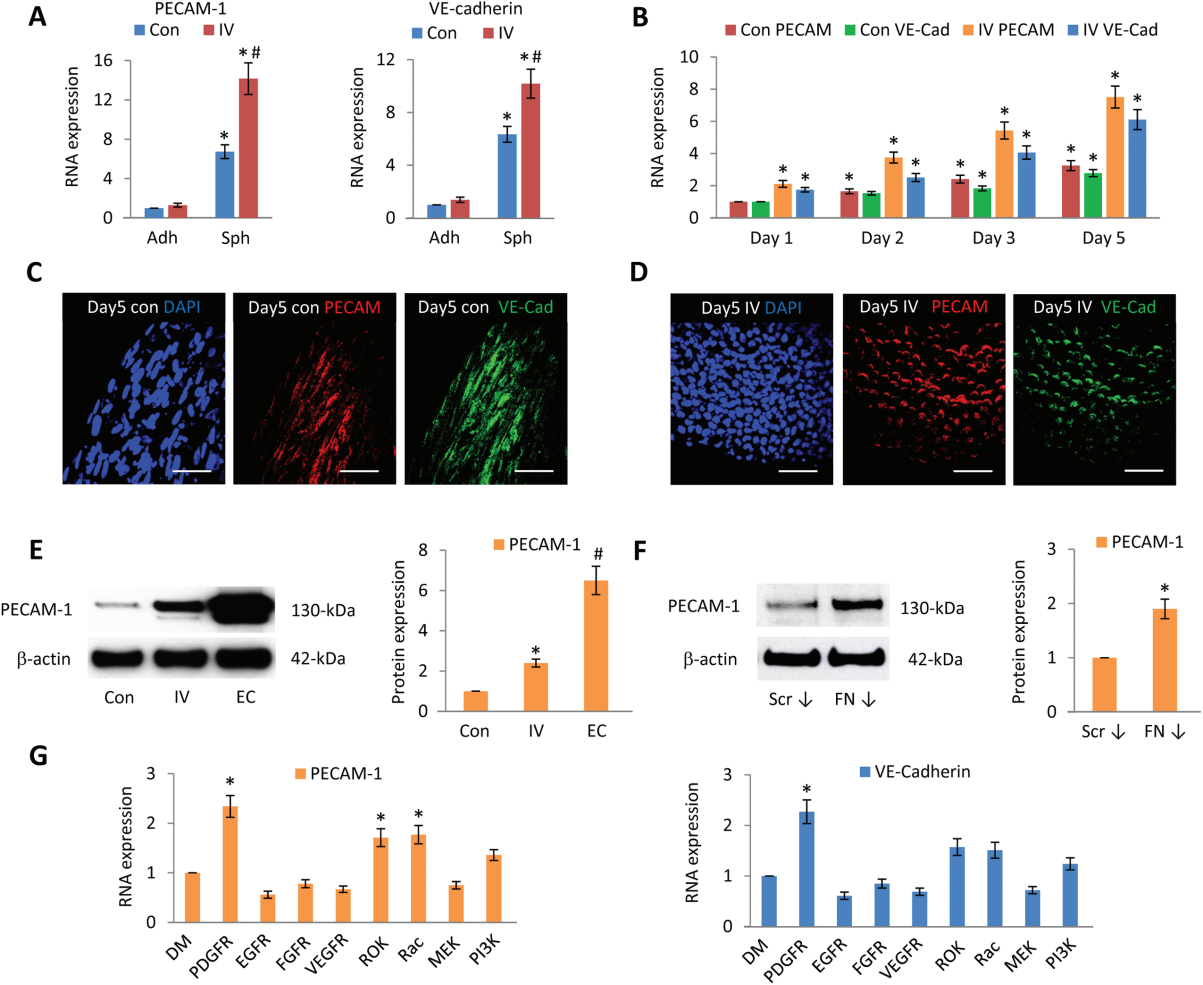
PDGFR and FN-inhibited spheroids upregulate endothelial markers. (A): Quantitative RT-PCR analysis of PECAM-1 and VE-cadherin expression within high-density adherent mesenchymal stromal/stem cells (MSCs) (Adh Con), high-density adherent MSCs exposed to PDGFR inhibitor-IV (Adh IV), control spheroids (Sph Con), and PDGFR inhibitor-IV spheroids (Sph IV), cultured for 5 days. Data are relative to GAPDH and normalized to adherent control levels. *, *p* < .001 compared with adherent controls, ^#^, *p* < .001 compared with control spheroids, using paired *t* test *n* = 3 separate experiments, error bars represent SD. (B): Quantitative RT-PCR analysis of PECAM-1 and VE-cadherin expression within control spheroids (Con) and PDGFR inhibitor-IV spheroids (IV), cultured for 1, 2, 3, and 5 days. Data are relative to GAPDH and normalized to the level of control spheroids at day 1. *, *p* < .001 compared with day 1 control spheroids, using paired *t* test *n* = 3 separate experiments, error bars represent SD. (C, D): Whole mount immunofluorescence analysis of (C) control spheroids (con) and (D) PDGFR inhibitor-IV spheroids (IV), cultured for 5 days, showing PECAM-1 (red) and VE-cadherin (green) expression, with DAPI-stained nuclei (blue). Scale bars = 50 µm. (E, F): Immunoblot analysis of PECAM-1 expression within control (Con), PDGFR inhibitor-IV (IV) spheroids, HUVECs (EC) and control scrambled siRNA spheroids (Scr ↓), and FN knockdown spheroids (FN ↓) after 5 days culture, with β-actin as loading control. Histogram shows PECAM-1 expression relative to β-actin and normalized to control spheroid or control siRNA spheroid levels. *, *p* < .001 compared with control spheroids, ^#^, *p* < .001 compared with PDGFR inhibitor-IV spheroids, using paired *t* test *n* = 3 separate experiments, error bars represent SD. (G): Quantitative RT-PCR analysis of PECAM-1 and VE-cadherin expression within control spheroids, cultured for 5 days in the presence of DMSO (DM) carrier, or 0.1 µM PDGFR inhibitor-IV, 2 nM EGFR, 0.1 µM FGFR, 0.5 µM VEGFR, 5 nM ROK, 50 µM Rac1, 20 µM MEK, or 5 µM PI3K inhibitors. Data are relative to GAPDH and normalized to DMSO treated spheroid levels. *, *p* < .001 compared with control spheroids, using paired *t* test *n* = 3 separate experiments, error bars represent SD. Abbreviations: FN, fibronectin; PDGFR, platelet-derived growth factor receptor.

Compared to untreated adherent MSCs, control spheroids expressed significantly increased PECAM-1 (6.7 ± 0.7-fold) and VE-cadherin (6.4 ± 0.6-fold) transcript levels (Fig. 4A). However, compared to control spheroids, PDGFR inhibitor-IV spheroids expressed an even higher level of PECAM-1 (2.1 ± 0.23-fold) and VE-cadherin (1.6 ± 0.17-fold) (Fig. 4A). Control spheroids increased the level of both PECAM-1 and VE-cadherin transcripts up to day 5, while PDGFR inhibitor-IV spheroids produced a greater increase (Fig. 4B). Immunofluorescence analysis revealed that control spheroids, which contained spindle-shaped MSCs, expressed both PECAM-1 and VE-cadherin protein, which increased up to day 5 (Fig. 4C and Supporting Information Fig. S4A). Similarly, PDGFR inhibitor-IV spheroids, which contained more rounded MSCs, displayed prominent PECAM-1 and VE-cadherin expression, but in this case the protein appeared localized around individual cells (Fig. 4D and Supporting Information Fig. S4B). Compared to control spheroids at day 5, PDGFR inhibitor-IV spheroids significantly expressed a higher level of PECAM-1 protein (2.4 ± 0.2-fold) (Fig. 4E), which was JAK signaling dependent (Supporting Information Fig. S2D–S2F). Compared to PDGFR inhibitor-IV spheroids, human umbilical vein endothelial cells (HUVECs) expressed a greater level of PECAM-1 (2.7 ± 0.4-fold).

FN knockdown spheroids, which caused similar effects on cell shape and phenotype (Fig. 1), increased PECAM-1 protein (1.9 ± 0.17-fold) (Fig. 4F). The effects of EGFR, FGFR, VEGFR, ROK, Rac, MEK, or PI3K signaling inhibitors on PECAM-1 and VE-cadherin transcripts were also examined, but only ROK or Rac inhibition markedly increased PECAM-1 expression (Fig. 4G). Rac or ROK-inhibited spheroids were shown to increase PECAM-1 protein (1.63 ± 0.13-fold and 1.68 ± 0.15-fold, respectively) (Supporting Information Fig. S4C); however, this level was lower than that expressed by PDGFR inhibitor-IV or FN-depleted spheroids.

### MSC Spheroids Secrete Angiogenic Factors

**Figure 5:**
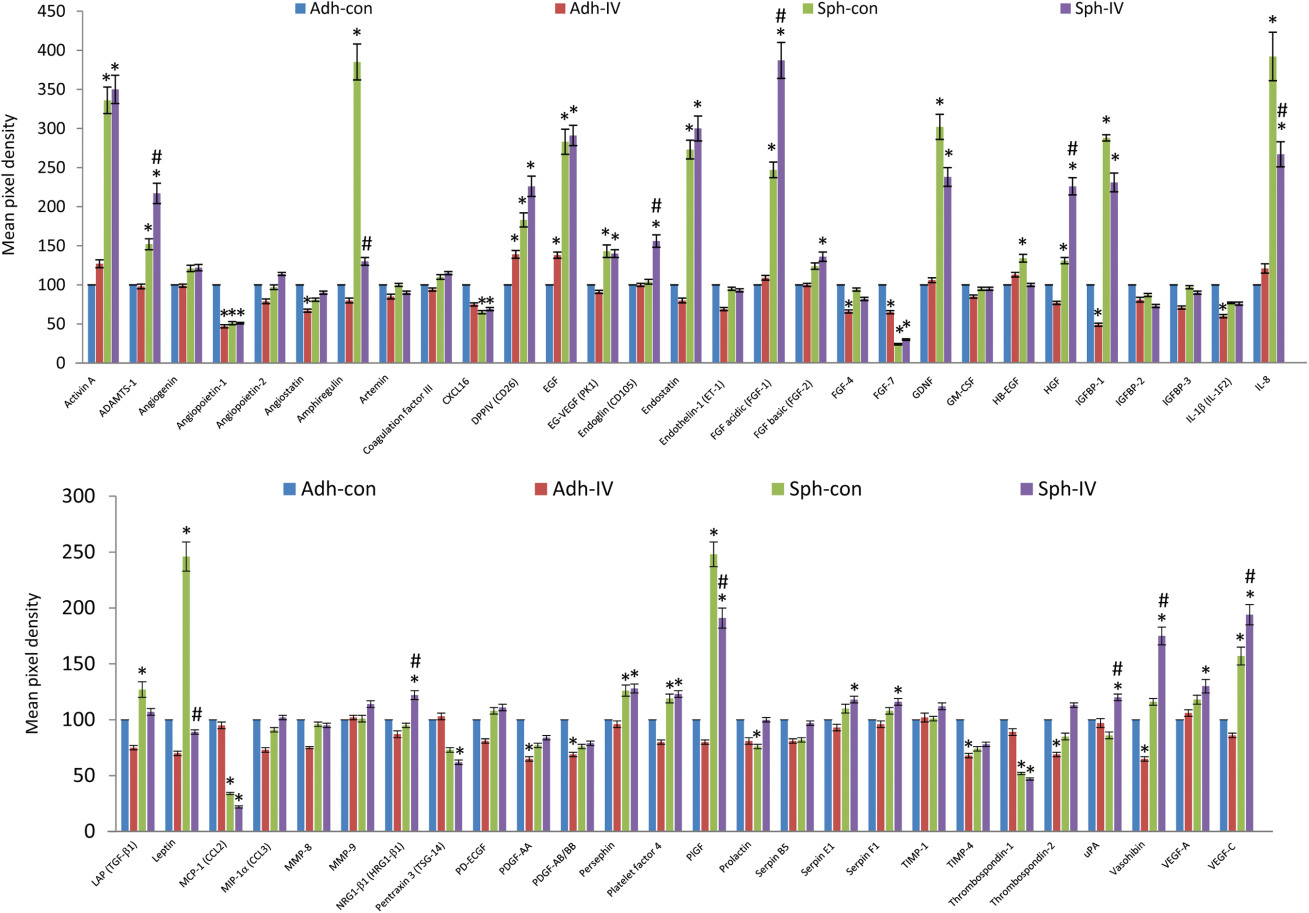
Angiogenic factors secreted by mesenchymal stromal/stem cell (MSC) spheroids. A human angiogenesis array kit (ARY007) (R&D Systems, Minneapolis, MN, http://www.rndsystems.com) was used to determine simultaneously the relative expression levels of 55 different angiogenic proteins, secreted by high-density adherent MSCs (Adh Con), high-density adherent MSCs exposed to PDGFR inhibitor-IV (Adh IV), control spheroids (Sph Con), and PDGFR inhibitor-IV spheroids (Sph IV), cultured for 3 days. Prior to analysis, adherent MSCs and spheroids were cultured for 2 days, then fresh growth medium added and cultured for a further 3 days for analysis. Supernatants from 24 identical adherent or spheroid cultures were pooled for analysis. Data are normalized to the DNA content of each MSC culture, and relative to high-density adherent MSCs (Adh con) *, *p* < .001 compared with adherent controls, ^#^, *p* < .005 compared with control spheroids, using paired *t* test *n* = 1 experiment, error bars represent SD between two repeats.

To identify proteins secreted by MSC spheroids which may regulate the endothelial features identified, we used a human angiogenesis proteome array to analyze supernatants from adherent 2D MSCs at high density, or 3D MSC spheroids, cultured in the presence or absence of PDGFR inhibitor-IV for 3 days (Fig. 5).

Compared to adherent controls, both control and PDGFR-IV spheroids prominently upregulated the secretion of activin-A, ADAMTS-1, dipeptidyl peptidase-4, EGF, endostatin, FGF-1, glial cell line-derived neurotrophic factor, IGFBP-1, IL-8, PlGF, and VEGF-C, but downregulated the secretion of angiopoietin-1, FGF-7, monocyte chemotactic protein-1, and thrombospondin-1. Compared to control spheroids, PDGFR-IV spheroids secreted higher levels of ADAMTS-1 (1.5 ± 0.12-fold), endoglin (1.5 ± 0.13-fold), FGF-1 (1.6 ± 0.11-fold), HGF (1.7 ± 0.13-fold), vasohibin (1.5 ± 0.12-fold), and VEGF-C (1.3 ± 0.09-fold), but markedly decreased the secretion of amphiregulin (3.9 ± 0.3-fold), IL-8 (1.5 ± 0.12-fold), and leptin (2.5 ± 0.2-fold). A list of the relative expression levels of all 55 secreted proteins is given in Supporting Information Table S3.

Taken together, these data demonstrate that the 3D culture of MSCs as spheroids markedly increased the secretion of potent angiogenic-related proteins, and dramatically upregulated the expression of PECAM-1 and VE-cadherin transcripts, compared with 2D culture of MSCs at high density. Furthermore, PDGFR inhibitor-IV and FN-depleted spheroids displayed an increase in PECAM-1 protein when compared with control spheroids, indicating that inhibition of mesenchymal drivers is a primary determinant in the acquisition of these endothelial features.

### MSC Spheroids Facilitate Blood Vessel Formation Within Matrigel

**Figure 6:**
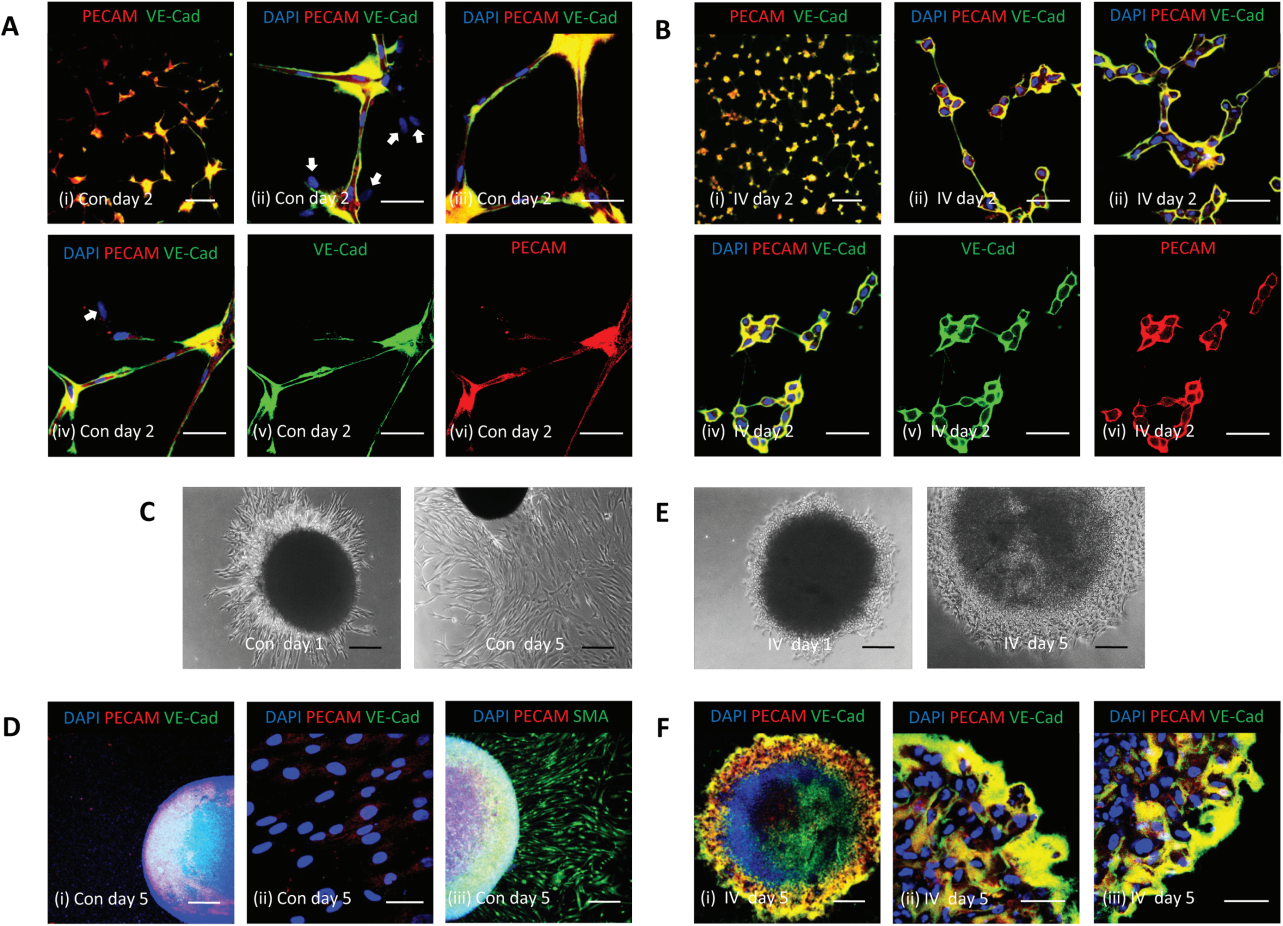
Spheroid-derived platelet-derived growth factor receptor (PDGFR)-inhibited mesenchymal stromal/stem cells (MSCs) form Matrigel-induced networks. (A, B): Immunofluorescence analysis of MSCs cultured as spheroids for 5 days, then dissociated into single cells (2 × 10^4^) and cultured on glass coverslips (13-mm diameter) coated with a thin layer of growth factor reduced Matrigel for a further 2 days. (A) MSCs derived from control spheroids (Con) and (B) MSCs derived from PDGFR inhibitor-IV spheroids (IV), showing network formation and PECAM-1 (red) and VE-cadherin (green) expression, with DAPI-stained nuclei (blue). Image (iv) is shown split into individual channels (v) green (VE-cadherin) and (vi) red (PECAM-1). Arrows in images A (ii, iv) represent MSCs lacking either PECAM-1 or VE-cadherin. Scale bars = 200 µm (i) and 50 µm (ii-vi). (C, E): Bright-field images of (C) control spheroids (Con) and (E) PDGFR inhibitor-IV spheroids (IV), implanted into Matrigel and cultured for 1 day and 5 days. Scale bars = 200 µm. (D, F): Whole mount immunofluorescence analysis of (D) control spheroids (Con) and (F) PDGFR inhibitor-IV spheroids (IV), implanted into Matrigel and cultured for 5 days, showing PECAM-1 (red) and VE-cadherin (green) expression and (D (iii)) SMA (green) expression, with DAPI-stained nuclei (blue). Scale bars = 200 µm (i) (D (iii)) and 50 µm (ii) (F (iii)).

Endothelial cells form capillary-like network structures when cultured on Matrigel 36. We therefore tested the ability of MSCs which had been cultured as spheroids, to form similar Matrigel-induced networks. Control and PDGFR inhibitor-IV spheroids were cultured for 5 days, then MSCs dissociated into single cells and cultured on Matrigel under identical conditions (i.e., no PDGFR inhibitor-IV added) for a further 2 days. PECAM-1 and VE-cadherin expressions were then determined by immunofluorescence analysis (Fig. 6A, 6B). MSCs derived from control spheroids readily established network structures with elongated branch points. These networks were composed of spindle-shaped cells which expressed both PECAM-1 and VE-cadherin (Fig. 6A). However, some cells expressed neither PECAM-1 nor VE-cadherin (Fig. 6A (ii, iv)). Similarly MSCs derived from PDGFR inhibitor-IV spheroids also readily formed widespread network structures, but in contrast, these had short branch points and the networks were composed of rounded cells, surrounded by distinctive PECAM-1 and VE-cadherin expression (Fig. 6B). Notably in this case, from >25 different images captured, every cell was positive for both PECAM-1 and VE-cadherin (Fig. 6B (ii-iv)).

As a prelude to examining the effects of MSC spheroids on angiogenesis *in vivo*, we cultured MSC spheroids within a 3D Matrigel plug *in vitro*. Control and PDGFR inhibitor-IV spheroids were cultured for 5 days. The intact spheroids were then implanted into Matrigel and cultured under identical conditions (i.e., no PDGFR inhibitor IV added) for a further 5 days. Control spheroids rapidly developed outgrowths of spindle-shaped cells by day 1, which became extensive by day 5 (Fig. 6C). These cellular outgrowths were negative for PECAM-1 and VE-cadherin (Fig. 6D (i-ii)), but positive for SMA (Fig. 6D (iii)). In contrast, PDGFR inhibitor-IV spheroids developed a ring of nonmigratory rounded cells around the spheroid periphery (Fig. 6E), which exhibited abundant PECAM-1 and VE-cadherin expression (Fig. 6F). Live cell imaging was also used to monitor the formation of cellular outgrowths from control spheroids and the peripheral ring of cells around PDGFR inhibitor-IV spheroids, during the first 90 hours of culture within Matrigel (Supporting Information Video S2).

### PDGFR-Inhibited MSCs Integrate with Functional In Vivo Blood Vessels

**Figure 7:**
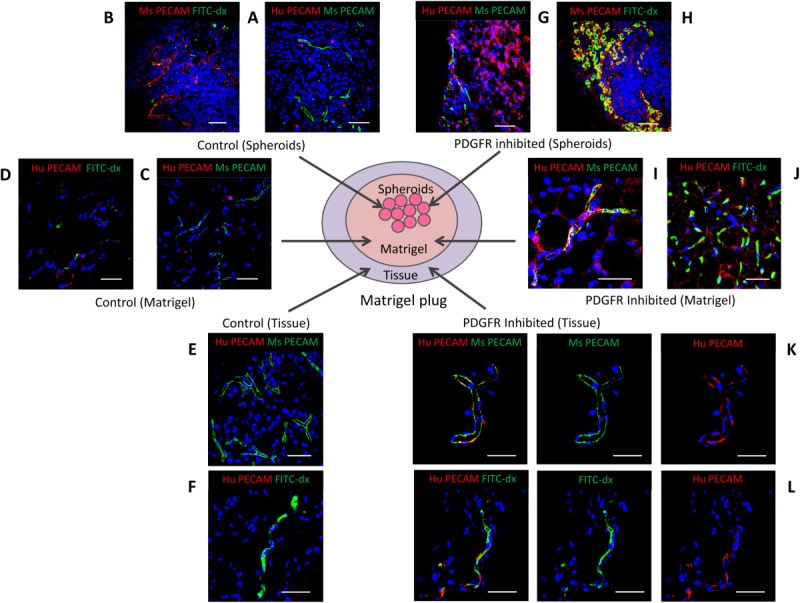
Spheroid-derived platelet-derived growth factor receptor (PDGFR)-inhibited mesenchymal stromal/stem cells integrate with perfused blood vessels. (A–L): Immunofluorescence analysis of Matrigel plugs containing control or PDGFR inhibitor-IV spheroids, after implantation in mice for 14 days, showing human (Hu) PECAM-1 (red) and murine (Ms) PECAM-1 (green) expression, or FITC-dextran (FITC-dx) perfusion (green), with DAPI-stained nuclei (blue). Images show representative areas of (A, B) control or (G, H) PDGFR inhibitor-IV spheroids. Matrigel surrounding (C, D) control or (I, J) PDGFR inhibitor-IV spheroids. Tissue surrounding (E, F) control or (K, L) PDGFR inhibitor-IV spheroids. Images K and L are shown split into their red channel (human PECAM-1) and green channel (murine PECAM-1 or FITC-dextran perfusion). Scale bars = 50 µm.

To determine the effects of MSC spheroids on *in vivo* angiogenesis, 10 control or PDGFR inhibitor-IV spheroids were suspended in Matrigel without any additional growth factors, and implanted into mice for 14 days, then human and murine PECAM-1 expression determined by immunofluorescence (Fig. 7). In addition, new functional blood vessels connected to the circulation were identified by FITC-dextran perfusion.

Examination of excised Matrigel plugs revealed that the control spheroids contained few human PECAM-1 positive cells present (Fig. 7A), but these spheroids were infiltrated by murine PECAM-1 positive blood vessels; the absence of FITC-dextran staining suggested that these vessels were not attached to the circulation (Fig. 7B). Similarly, the Matrigel surrounding the control spheroids was permeated with murine PECAM-1 positive blood vessels; however, few human PECAM-1-positive cells were observed (Fig. 7B), and these vessels were also not associated with FITC-dextran perfusion (Fig. 7D). Analysis of the peripheral tissue revealed numerous blood vessels (Fig. 7C), but only minimal human PECAM-1-positive staining was detected (Fig. 7D), which again was not detected near FITC-dextran staining (Fig. 7F).

In marked contrast, PDGFR inhibitor-IV spheroids contained numerous human PECAM-1-positive cells and these spheroids were also infiltrated by murine blood vessels (Fig. 7G), which were connected to the host circulation as indicated by abundant FITC-dextran perfusion (Fig. 7H). The Matrigel surrounding PDGFR inhibitor-IV spheroids was also permeated with both murine and human PECAM-1-positive cells, which associated together in vascular-like assemblies (Fig. 7I). Some of these human PECAM-1-positive vascular-like assemblies within Matrigel were perfused with FITC-dextran (Fig. 7J). Human PECAM-1-positive cells derived from PDGFR inhibitor-IV spheroids were also detected in the peripheral tissue, where they clearly integrated into blood vessels with murine PECAM-1 positive cells (Fig. 7K). These human PECAM-1-positive vessels in tissue were connected to the host vasculature and perfused with FITC-dextran (Fig. 7L). Thus, PDGFR inhibitor-IV spheroids are a potent source and stimulant for neovascularization.

## Discussion

Mesenchymal cells make tissues by depositing and embedding themselves in an extracellular matrix and growth factor-rich microenvironment, which they remodel and maintain throughout life. Although this extrinsic niche dictates the behavior of MSCs, their therapeutic potential remains severely constrained by lack of mechanistic insight into how the niche controls their fate. Here, we have shown that disrupting the extracellular matrix molecule FN or the functionally linked PDGFR, which together regulate mesenchyme 22, converts MSCs rapidly from SMA-rich spindle-shaped contractile cells to rounded E-cadherin-rich cells. These cells exhibit enhanced expression of markers for pluripotency, mesendoderm, endoderm, and angiogenic markers, and display potent angiogenic behavior *in vivo*. Thus, blocking natural mesenchymal signals offers an effective strategy for reprogramming mesenchymal cells and for therapeutic revascularization.

We have developed a novel approach to modulate MSC fate that does not require the use of viral vectors or exogenous DNA. Using a spheroid MSC culture model that recapitulates physiological features of a 3D cellular environment and cell-cell interactions, MSCs were induced toward a more multipotent state. Untreated MSCs within spheroids retained their spindle-shape and mesenchymal character with a SMA-rich cytoskeleton and profuse FN matrix. Although culturing MSCs as 3D spheroids was sufficient to induce some upregulation of mesendodermal and endodermal markers, inhibition of PDGFRs or FN knockdown induced rapid cell rounding along with significant further induction of pluripotency markers Oct4A and Nanog, demonstrating that MSCs have the potential to revert to a premesenchymal state, which is accentuated when mesenchymal signals are inhibited. We have previously shown using 2D cultured MSCs that PDGFR signaling inhibition changes their shape and cell fate 28. In this study, depletion of the extracellular matrix component FN was also shown to modulate MSC shape and direct their fate, emphasizing the crucial role played by cell shape in MSC fate decisions. Furthermore, we were able to demonstrate that these cells had strong angiogenic potential *in vitro* and *in vivo*. Thus, disrupting mesenchymal signals in these 3D cultures induced a mesenchymoangioblast-like state 3,4.

It has been unclear whether MSCs possess the ability to transdifferentiate to functional endothelial cells; most reports have relied on exogenous VEGF-A supplementation approaches 8. We previously showed that high-density cultures exhibited Notch-dependent endothelial potential *in vitro* and in CAM assays 9. Here, by demonstrating that MSCs derived from PDGFR-inhibited spheroids are able to induce, and integrate with functional blood vessels perfused by the circulation *in vivo*, we have directly shown their angiogenic and vascular potential. By inhibiting mesenchymal signals, we were able to induce endothelial fate, including the reinstigation of robust cell-cell interactions, as judged by E-cadherin expression. Thus cell-cell contacts within PDGFR-inhibited spheroids may be just as important in driving the angiogenic features, as increased expression of embryonic transcription factors.

## Conclusion

In summary, we have shown that the mesenchymal fate of MSCs can be modulated by re-engineering the relationship between cells and their local matrix, without the need for viral delivery of exogenous transcription factors. By blocking mesenchymal drivers, these cells can be reverted to a mesenchymoangioblast-like state and thence to functional endothelial-like cells *in vivo*. As these strategies target the natural mechanisms that manipulate mesenchymal fate, they have great potential for future revascularization therapies.
