# Supplementary figures and images for: Mesenchymal Stromal Cells: Inhibiting PDGF Receptors or Depleting Fibronectin Induces Mesodermal Progenitors with Endothelial Potential
Source: Stem Cells. 2014 Feb 19;32(3):694–705. doi: 10.1002/stem.1538 (PMC4377076; doi:10.1002/stem.1538)

Fig. S1


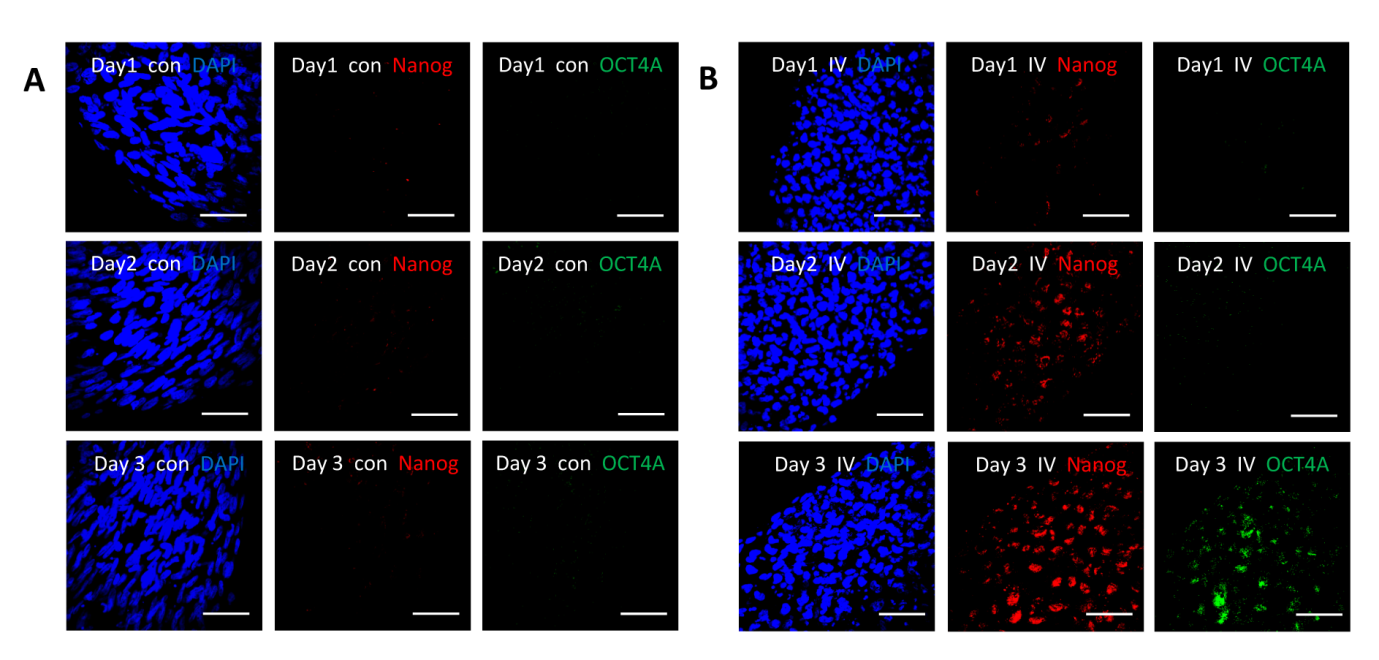


Fig. S2


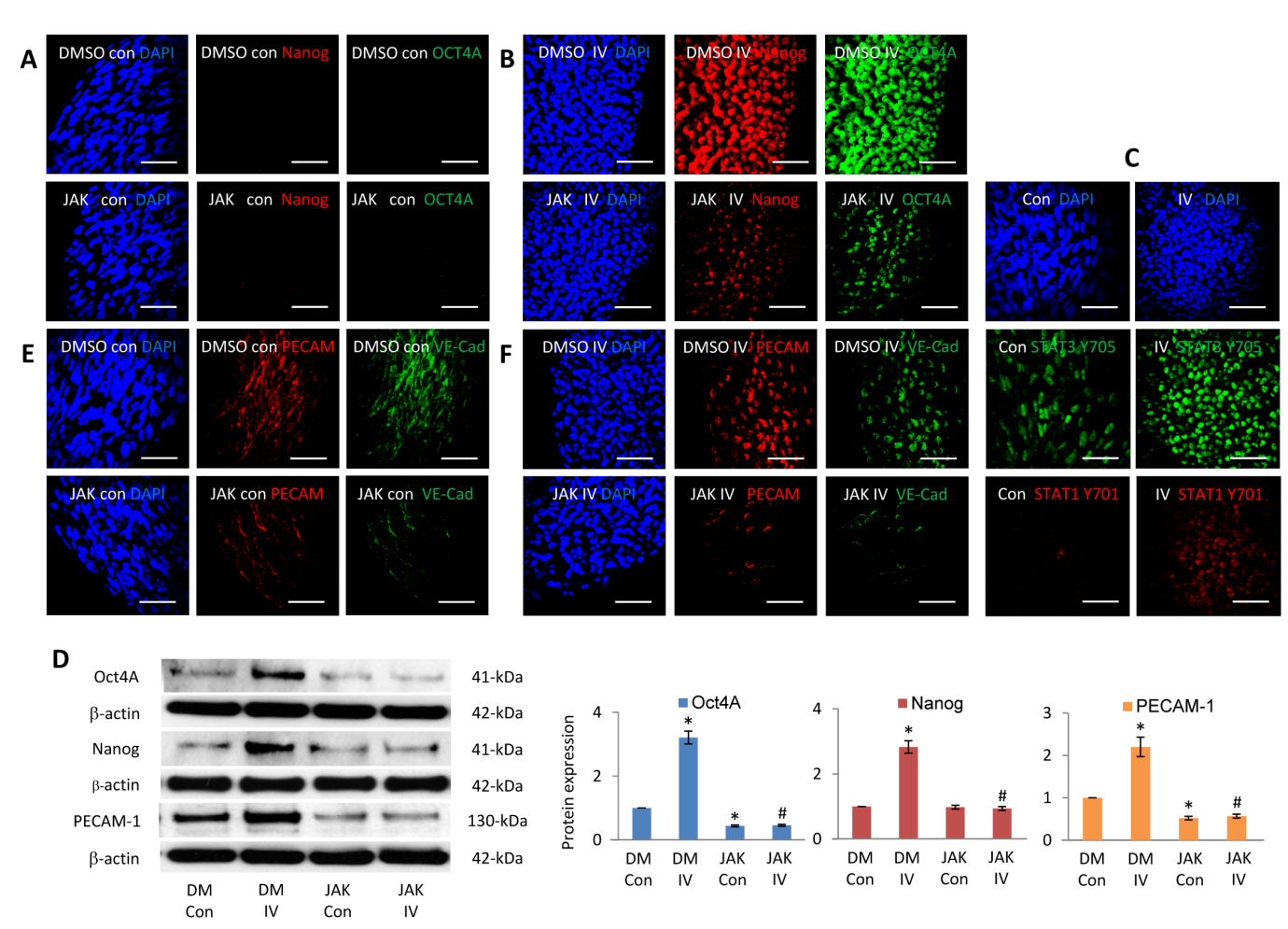


Fig. S3


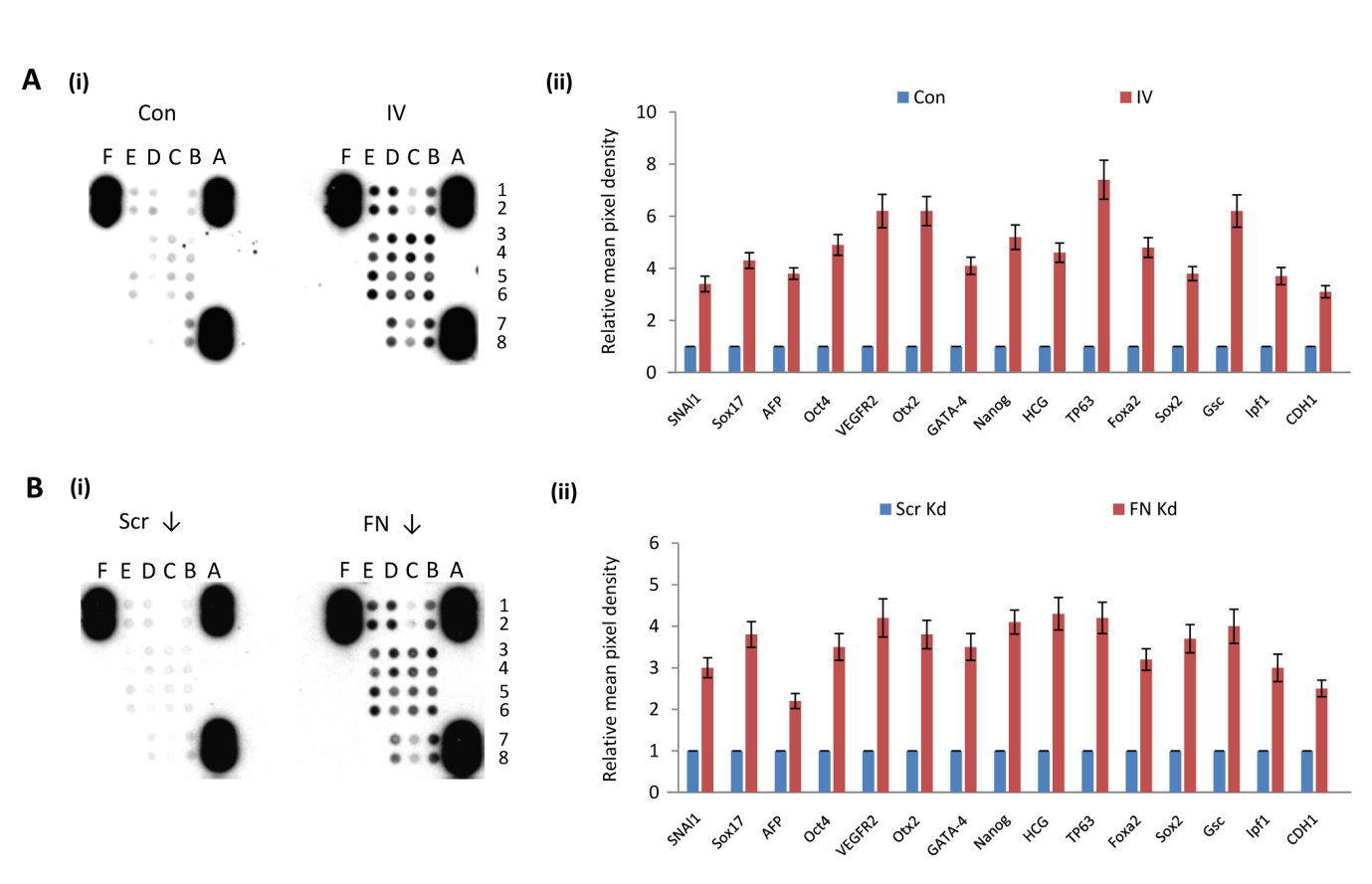


Fig. S4


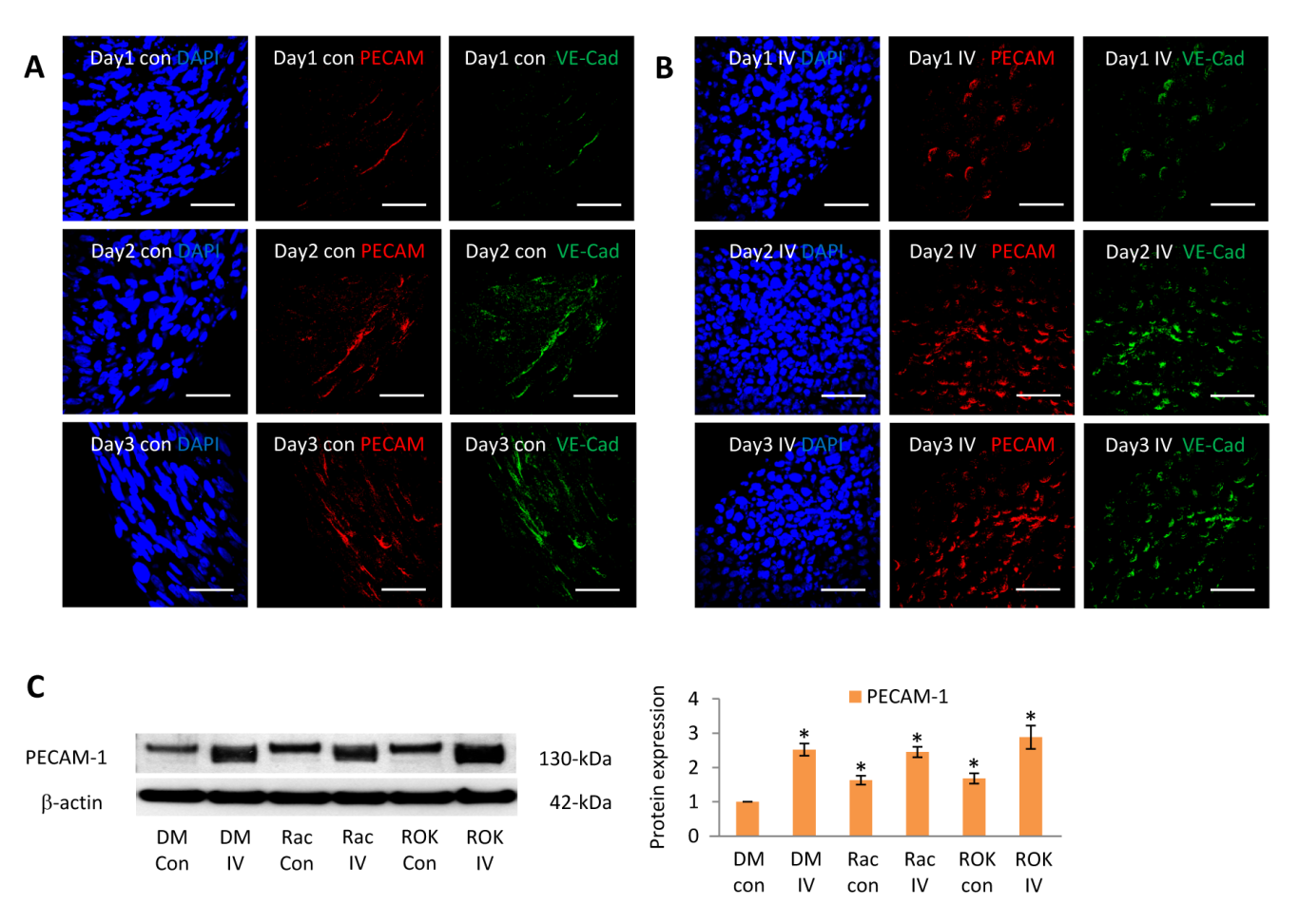

Supplement: Supplementary file 1 — Supporting Information Figures [file stem0032-0694-sd1.docx]
