# Supplementary material for: Mesenchymal Stromal Cells: Inhibiting PDGF Receptors or Depleting Fibronectin Induces Mesodermal Progenitors with Endothelial Potential
Source: Stem Cells. 2014 Feb 19;32(3):694–705. doi: 10.1002/stem.1538 (PMC4377076; doi:10.1002/stem.1538)
Supplement: Supplementary file 2 — Supporting Information Figure Legends [file stem0032-0694-sd2.doc]

**Supplementary figures**

Figure S1. PDGFR inhibited spheroids upregulate Oct4A and Nanog

**(A,B)** Whole mount immunofluorescence analysis of (**A**) control spheroids (Con) and (**B**) PDGFR inhibitor-IV spheroids (IV), cultured for 1, 2 and 3 days, showing Nanog (red) and Oct4A (green) expression, with DAPI-stained nuclei (blue). Scale bars = 50 μm.

Figure S2. Oct4A, Nanog and PECAM-1 expression are JAK-dependent

**(A,B)** Whole mount immunofluorescence analysis of (**A**) control spheroids (Con) and (**B**) PDGFR inhibitor-IV spheroids (IV), cultured for 5 days in the presence of DMSO carrier or 20 nM JAK inhibitor, showing Nanog (red) and Oct4A (green) expression, with DAPI-stained nuclei (blue). Scale bars = 50 μm.

**(C)** Whole mount immunofluorescence analysis of control spheroids (Con) and PDGFR inhibitor-IV spheroids (IV), cultured for 5 days, showing STAT3 Y705 (green) and STAT1 Y701 (red) expression, with DAPI-stained nuclei (blue). Scale bars = 50 μm.

**(D)** Immunoblot analysis of Oct4A, Nanog and PECAM-1 expression within control spheroids (Con) and PDGFR inhibitor-IV spheroids (IV), cultured for 5 days in the presence of DMSO (DM) carrier or 20 nM JAK inhibitor, with β-actin as a loading control. Histograms show Oct4A, Nanog and PECAM-1 expression relative to β-actin and normalized to DMSO treated control spheroid levels. *, *p* [lt] .001 compared with control spheroids, #, *p* [lt] .001 compared with PDGFR inhibitor-IV spheroids, using paired *t*-test n [mt] 3 separate experiments, error bars represent SD.

**(E,F)** Whole mount immunofluorescence analysis of (**E**) control spheroids (Con) and (**F**) PDGFR inhibitor-IV spheroids (IV), cultured for 5 days in the presence of DMSO carrier or 20 nM JAK inhibitor, showing PECAM-1 (red) and VE-cadherin (green) expression, with DAPI-stained nuclei (blue). Scale bars = 50 μm.

Figure S3. PDGFR inhibitor-IV and FN knockdown spheroids up-regulate a range of endoderm and pluripotent markers

**(A, B)** A human pluripotent stem cell array kit (ARY010) (R&D Systems), was used to determine simultaneously the relative expression levels of 15 different stem cell markers. Proteome array analysis of endoderm and pluripotent proteins expressed by (**A**) control spheroids (Con) and PDGFR inhibitor-IV spheroids (IV) or (**B**) scrambled control siRNA spheroids (Scr [darrow]) and FN knockdown spheroids (FN [darrow]), cultured for 5 days. Lysates from 24 identical spheroid cultures were pooled for analysis. (i) Proteome arrays; coordinates (A1,A2), (A7,A8) and (F1,F2) are reference spots, (B1,B2) Oct3/4, (B3,B4) Nanog, (B5,B6) Sox2, (B7,B8) CDH1, (C1,C2) AFP, (C3,C4) GATA-4, (C5,C6) Foxa2, (C7,C8) Ipf1, ((D1,D2) Sox17, (D3,D4) Otx2, (D5,D6) TP63, (D7,D8) Gsc, (E1,E2) SNAl1, (E3,E4) VEGFR2 and (E5,E6) HCG (ii) Histogram showing protein levels relative to control spheroids. Data is *n* = 1 experiment, error bars represent SD between two repeats.

Figure S4. DGFR, Rac1 or ROK inhibited spheroids up-regulate endothelial markers

**(A,B)** Whole mount immunofluorescence analysis of (**A**) control spheroids (con) and (**B**) PDGFR inhibitor-IV spheroids (IV), cultured for 1, 2 and 3 days, showing PECAM-1 (red) and VE-cadherin (green) expression, with DAPI-stained nuclei (blue). Scale bars = 50 μm.

**(C)** Immunoblot analysis of PECAM-1 expression within control spheroids (Con) and PDGFR inhibitor-IV spheroids (IV) cultured for 5 days in the presence of carrier DMSO (DM), or 50 μM Rac1 or 5 nM ROK inhibitors, with β-actin as a loading control. Histogram shows PECAM-1 expression relative to β-actin and normalized to DMSO treated control spheroid levels. *, *p* [lt] .001 compared with control spheroids, using paired *t*-test n [mt] 3 separate experiments, error bars represent SD.

**Supplemental online video 1. Assembly of spheroids**

Live cell image using a Cell-IQ cell imaging and analysis system (CM Technologies), monitoring 60,000 MSCs ± 0.1 μM PDGFR inhibitor-IV seeded into an individual well of a low cell binding 96-well plate and cultured at 37°C for 20 hours. Left image shows PDGFR inhibitor-IV spheroid, right image shows control spheroid.

**Supplemental online video 2. Outgrowths from implanted spheroids**

Live cell image using a Cell-IQ cell imaging and analysis system (CM Technologies), monitoring 5-day control and PDGFR inhibitor-IV spheroids implanted into Matrigel and cultured at 37°C for 90 hours. Left image shows PDGFR inhibitor-IV spheroid, right image shows control spheroid.
