# Supplementary material for: Mesenchymal Stromal Cells: Inhibiting PDGF Receptors or Depleting Fibronectin Induces Mesodermal Progenitors with Endothelial Potential
Source: Stem Cells. 2014 Feb 19;32(3):694–705. doi: 10.1002/stem.1538 (PMC4377076; doi:10.1002/stem.1538)
Supplement: Supplementary file 3 — Supporting Information [file stem0032-0694-sd3.docx]

**Table S1. Quantitative RT-PCR primer sequences**

| **Gene** | **Forward primer** | **Reverse primer** |
| --- | --- | --- |
| AFP | 5'-ACCTCGTCGGAGCTGATG-3' | 5'-TCGCCACAGGCCAATAGT-3' |
| CDH1 | 5'-AGGAGGCGGAGAAGAGGA-3' | 5'-GAGGGTTGGTGCAACGTC-3' |
| CDH2 | 5'-AATGACCCCACAGCTCCA-3' | 5'-GAGCTCAAGGACCCAGCA-3' |
| EOMES | 5'-TCAATCCCACTGCCCACT-3' | 5'-TGCCAGAGGTCACCCATT-3' |
| Fibronectin (FN1) | 5'-CCGGCCTGGAGTACAATG-3' | 5'-GCACCTCTGGGATGATGG-3' |
| FOXA1 | 5'-GACCCCAACCCCTTTGTC-3' | 5'-TGGCACTCTGCAAAGCAA-3' |
| FOXA2 | 5'-CCCGGTCACGAACAAAAC-3' | 5'-TAATGGGCCGGGAGTACA-3' |
| FOXH1 | 5'-CCCAGATCATCCGTCAGG-3' | 5'-AAGGTTGTGGCGAATGGA-3' |
| GAPDH | 5'-AAGGGCATCCTGGGCTAC-3' | 5'-GTGGAGGAGTGGGTGTCG-3' |
| GSC | 5'-CGCGAGGAGAAAGTGGAG-3' | 5'-GATGAGGACCGCTTCTGC-3' |
| MIXL1 | 5'-ACAGGCGTGCCAAGTCTC-3' | 5'-CGTTTCAGTTCCAGGAGCA-3' |
| NANOG | 5'-CCTCCTCCCATCCCTCAT-3' | 5'-GGATGGGCATCATGGAAA-3' |
| NODAL | 5'-AGTTGCTCTGCCCACCAG-3' | 5'-AGCAGCCTCTGTGCTTGG-3' |
| Oct4A (POU5F1) | 5'-CCCTCCAGGTGGTGGAGG-3' | 5'-GGCCTTGGAAGCTTAGCC-3' |
| PECAM1 | 5'-TGGCGCATGCCTGTAGTA-3' | 5'-TCCGTTTCCTGGGTTCAA-3' |
| SM α-actin (ACTG2) | 5'-CATCACCAACTGGGACGA-3' | 5'-GGTGGGATGCTCTTCAGG-3' |
| SOX17 | 5'-GCAAGATGCTGGGCAAGT-3' | 5'-CTCTGCCTCCTCCACGAA-3' |
| VE-cadherin (CDH5) | 5'-GGAGCCGAGCATGTGTCT-3' | 5'-TCTGCAAGGTGTGCCTGA-3' |
| VIM | 5'-GCCAACCGGAACAATGAC-3' | 5'-GGGCATCCACTTCACAGG-3' |

**Table S2. Details of antibodies**

| **Antibody** | **Supplier** | **Application** |
| --- | --- | --- |
| β-actin (A1978)  mouse anti-human | Sigma-Aldrich | Immunoblotting (1:5000) |
| CDH1 (#3195)  Rabbit anti-human | Cell Signaling Technology | Immunofluorescence (1:100) |
| Fibronectin (F3648)  Rabbit anti-human | Sigma-Aldrich | Immunoblotting (1:1000)  Immunofluorescence (1:200) |
| Nanog (AF1997)  Goat anti-human | R&D Systems | Immunoblotting (1:200) Immunofluoresence (1:50) |
| Oct4 (sc-5279)  Mouse anti-human | Santa Cruz Biotechnology | Immunoblotting (1:200) Immunofluoresence (1:50) |
| PDGFRβ (sc-339)  Rabbit anti-human | Santa Cruz Biotechnology | Immunoblotting (1:1000) |
| PDGFRβ (Y1021) (sc-12909)  Rabbit anti-human | Santa Cruz Biotechnology | Immunoblotting (1:1000) |
| PDGFRβ (Y751) (sc-21902)  Rabbit anti-human | Santa Cruz Biotechnology | Immunoblotting (1:1000) |
| PECAM-1 (sc-1506)  Rabbit anti-human | Santa Cruz Biotechnology | Immunoblotting (1:500)  Immunofluorescence (1:100) |
| PECAM-1 (sc-8306)  Rabbit anti-human | Santa Cruz Biotechnology | *In vivo* Immunofluorescence (1:100) |
| PECAM-1 (#557355)  Rat anti-mouse | BD Bioscience | *In vivo* Immunofluorescence (1:100) |
| SM α-actin (M0851)  Mouse anti-human | Dako | Immunoblotting (1:1000)  Immunofluorescence (1:200) |
| STAT1 (Y701) (#9167)  Rabbit anti-human | Cell Signaling Technology | Immunofluorescence (1:400) |
| STAT3 (Y705) (#9131)  Mouse anti-human | Cell Signaling Technology | Immunofluorescence (1:100) |
| VE-cadherin (sc-52751)  Mouse anti-human | Santa Cruz Biotechnology | Immunofluorescence (1:100) |

**Table S3. Relative expression levels of secreted angiogenic proteins**

| **Protein** | **Adh-con** | **Adh-IV** | **Sph-con** | **Sph-IV** |
| --- | --- | --- | --- | --- |
| Activin-A | 100 | 127 ± 6 | 336 ± 21 | 350 ± 23 |
| ADAMTS-1 | 100 | 98 ± 5 | 152 ± 11 | 217 ± 16 |
| Angiogenin | 100 | 99 ± 5 | 121 ± 7 | 122 ± 5 |
| Angiopoietin-1 | 100 | 47± 2 | 51 ± 3 | 51 ± 2 |
| Angiopoietin-2 | 100 | 79 ± 4 | 97 ± 5 | 114 ± 7 |
| Angiostatin | 100 | 67 ± 3 | 81 ± 4 | 90 ± 5 |
| Amphiregulin | 100 | 80 ± 4 | 385 ± 23 | 131 ± 6 |
| Artemin | 100 | 85 ± 3 | 100 ± 5 | 90 ± 4 |
| Coagulation factor III | 100 | 94 ± 5 | 111 ± 6 | 115 ± 5 |
| CXCL16 | 100 | 76 ± 4 | 65 ± 4 | 69 ± 3 |
| DPPIV (CD26) | 100 | 139 ± 7 | 183 ± 12 | 226 ± 15 |
| EGF | 100 | 139 ± 6 | 284 ± 19 | 291 ± 20 |
| EG-VEGF (PK1) | 100 | 91 ± 5 | 144 ± 8 | 140 ± 7 |
| Endoglin | 100 | 100 ± 5 | 104 ± 5 | 156 ± 8 |
| Endostatin | 100 | 81 ± 4 | 274 ± 17 | 301 ± 21 |
| Endothelin-1 | 100 | 69 ± 3 | 95 ± 5 | 93 ± 5 |
| FGF-1 | 100 | 109 ± 5 | 247 ± 15 | 387 ± 23 |
| FGF-2 | 100 | 100 ± 4 | 124 ± 6 | 136 ± 7 |
| FGF-4 | 100 | 66 ± 3 | 94 ± 4 | 82 ± 4 |
| FGF-7 | 100 | 65 ± 3 | 24 ± 2 | 30 ± 2 |
| GDNF | 100 | 106 ± 5 | 302 ± 18 | 238 ± 15 |
| GM-CSF | 100 | 85 ± 4 | 95 ± 5 | 95 ± 4 |
| HB-EGF | 100 | 113 ± 6 | 134 ± 7 | 100 ± 5 |
| HGF | 100 | 77 ± 4 | 131 ± 6 | 226 ± 13 |
| IGFBP-1 | 100 | 49 ± 2 | 288 ± 18 | 231 ± 14 |
| IGFBP-2 | 100 | 81 ± 4 | 87 ± 4 | 73 ± 3 |
| IGFBP-3 | 100 | 71 ± 3 | 97 ± 5 | 90 ± 4 |
| IL-1β | 100 | 60 ± 3 | 77 ± 4 | 76 ± 3 |
| IL-8 | 100 | 121 ± 6 | 392 ± 31 | 265 ± 18 |
| LAP (TGF-β1) | 100 | 75 ± 3 | 127 ± 7 | 107 ± 6 |
| Leptin | 100 | 70 ± 4 | 246 ± 15 | 89 ± 4 |
| CCL2 | 100 | 95 ± 5 | 34 ± 3 | 22 ± 2 |
| CCL3 | 100 | 73 ± 3 | 91 ± 5 | 102 ± 6 |
| MMP-8 | 100 | 75 ± 4 | 96 ± 5 | 95 ± 4 |
| MMP-9 | 100 | 102 ± 5 | 101 ± 5 | 114 ± 6 |
| NRG1-β1 (HRG1-β1) | 100 | 87 ± 4 | 95 ± 5 | 122 ± 6 |
| Pentraxin 3 (TSG-14) | 100 | 103 ± 5 | 73 ± 4 | 63 ± 3 |
| PD-ECGF | 100 | 81 ± 4 | 108 ± 6 | 111 ± 7 |
| PDGF-AA | 100 | 65 ± 4 | 77 ± 4 | 84 ± 5 |
| PDGF-AB/BB | 100 | 69 ± 4 | 76 ± 4 | 79 ± 4 |
| Persephin | 100 | 96 ± 5 | 126 ± 7 | 128 ± 6 |
| Platelet factor 4 | 100 | 80 ± 4 | 119 ± 6 | 123 ± 8 |
| PlGF | 100 | 81 ± 4 | 248 ± 17 | 191 ± 15 |
| Prolactin | 100 | 80 ± 4 | 76 ± 3 | 100 ± 5 |
| Serpin B5 | 100 | 81 ± 4 | 82 ± 3 | 97 ± 5 |
| Serpin E1 | 100 | 93 ± 4 | 110 ± 5 | 118 ± 5 |
| Serpin F1 | 100 | 96 ± 5 | 108 ± 5 | 116 ± 6 |
| TIMP-1 | 100 | 102 ± 5 | 101 ± 5 | 112 ± 5 |
| TIMP-4 | 100 | 68 ± 4 | 74 ± 5 | 78 ± 5 |
| Thrombospondin-1 | 100 | 89 ± 5 | 52 ± 3 | 47 ± 3 |
| Thrombospondin-2 | 100 | 69 ± 4 | 85 ± 4 | 113 ± 6 |
| uPA | 100 | 97 ± 5 | 86 ± 4 | 120 ± 7 |
| Vasohibin | 100 | 65 ± 3 | 116 ± 5 | 175 ± 11 |
| VEGF-A | 100 | 106 ± 5 | 118 ± 5 | 130 ± 8 |
| VEGF-C | 100 | 86 ± 4 | 157 ± 9 | 194 ± 14 |
